# Localization of new peptidoglycan at poles in *Bacillus mycoides,* a member of the *Bacillus cereus* group

**DOI:** 10.1007/s00203-012-0830-1

**Published:** 2012-07-08

**Authors:** Luana Turchi, Tiziana Santini, Elena Beccari, Carmen Di Franco

**Affiliations:** 1 Dipartimento di Biologia e Biotecnologie “C. Darwin”, Università Sapienza, Roma, P.le A. Moro 5, 00185 Rome, Italy; 2 Istituto di Biologia e Patologia Molecolari IBPM-CNR, Università Sapienza, Roma, P.le A. Moro 5, 00185 Rome, Italy

**Keywords:** *B. mycoides*, *B. cereus* group, Cell wall, Peptidoglycan, Cell poles, Vancomycin staining

## Abstract

*Bacillus mycoides* is a sporogenic Gram-positive soil bacillus of the *B. cereus* group. This bacillus, which forms hyphal colonies, is composed of cells connected in filaments that make up bundles and turn clock- or counterclockwise depending on the strain. A thick peptidoglycan wall gives the rod cells of these bacilli strength and shape. One approach used to study peptidoglycan neoformation in Gram positives exploits the binding properties of antibiotics such as vancomycin and ramoplanin to nascent peptidoglycan, whose localization in the cell is monitored by means of a fluorescent tag. When we treated *B. mycoides* strains with BODIPY-vancomycin, we found the expected accumulation of fluorescence at the midcell septa and localization along the cell sidewall in small foci distributed quite uniformly. Intense fluorescence was also observed at the poles of many cells, more clearly visible at the outer edges of the cell chains. The unusual abundance of peptidoglycan intermediates at the cell poles after cell separation suggests that the construction process of this structure is different from that of *B. subtilis,* in which the free poles are rarely reactive to vancomycin.

## Introduction

The thick cell wall of Gram-positive bacilli contains several layers of peptidoglycan (PG), a unique giant polymer of glycan strands made of alternating *N*-acetylglucosamine and *N*-acetylmuramic acid, cross-linked by peptide bridges (Vollmer et al. 2008). The PG precursors are the lipid II GlcNAc-MurNAc-pentapeptides, linked to the membrane through the lipid anchor in the cellular sites of active PG synthesis (de Kruijff et al. 2008). The PG precursors and the pentapeptides of the nascent glycan chains expose a free D-Ala-D-Ala group specifically bound by the antibiotic vancomycin. When linked to different fluorochromes, vancomycin can be used to label, and hence visually detect, the spatial pattern of new PG localization within the cell. This approach has been used to detect the sites of cell wall neoformation in *B. subtilis* (Daniel and Errington 2003; Tiyanont et al. 2006), though no such information is available for the numerous bacilli with a similar cell shape that constitute the *B. cereus* group (Guinebretiere et al. 2008).

**Fig. 1:**
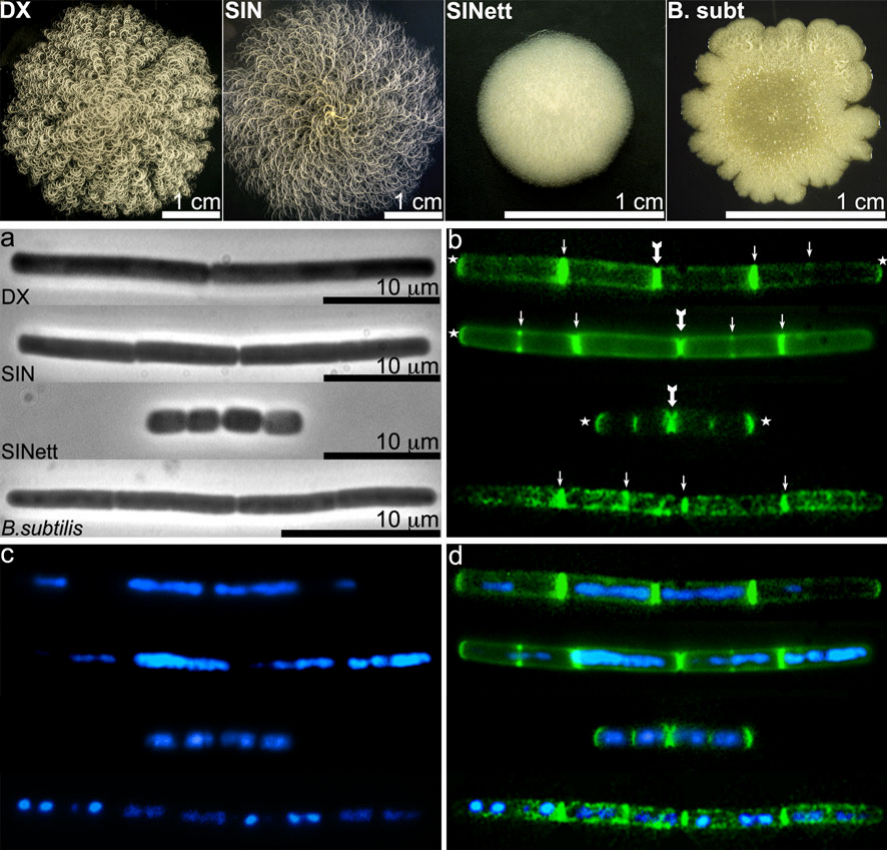
Colony shape and cells of *B. mycoides* DX, SIN, SINett and *B. subtilis* 168. *Top panel*: colonies of *B. mycoides* DX, SIN and SINett were grown on TS agar for 72 h at 30 °C, while *B. subtilis* colonies were grown at 37 °C. Note the macroscopic hyphal pattern and the opposite direction of filament bundles in wild-type *B. mycoides* strains (EMBO Journal published this *B. mycoides* DX colony image on the cover of volume 28, 16 Sept. 2009). *Bottom panel*: *B. mycoides* DX, SIN, SINett and *B. subtilis* 168 cells in the exponential growth phase were treated with Van-BDP/vancomycin to stain new PG and with DAPI to visualize nucleoids as described in “Materials and methods”. **a** Phase contrast images. **b** Van-BDP fluorescence: *arrows* point to septa, *thick arrows* to internal newly formed poles, *stars* to fluorescent free poles. Note that fluorescence along the lateral cell wall is localized in small foci in *B. mycoides* and in large foci in *B. subtilis*. The external poles of the *B. mycoides* filaments are often brightly fluorescent, while they are rarely decorated by Van-BDP in *B. subtilis*. **c** DAPI staining of nucleoids. See multiple nucleoids in DX and SIN cells. **d** Overlay of Van-BDP (*green*) and DAPI (*blue*). Images were merged using Adobe Photoshop version 7.0

*B. mycoides* is the member in this group that has received least attention because it is not as harmful to humans as *B. anthracis* and *B. cereus* or an insecticidal agent as *B. thuringiensis*. *B. mycoides* was first studied by the Russian naturalist Gause (1939), who investigated the genesis of the typical colonies of these soil bacilli, whose growth on agar plates forms an attractive rhizoidal shape resulting from cells connected at the poles that form long filaments resembling fungal hyphae, grouped in bundles curving to the right or to the left in different strains called dextral (DX) and sinistral (SIN) (Fig. 1 top panel). The strong cell-to-cell connections yield a stable network that favors interactions with other soil inhabitants, such as microbes, fungi and plant roots (Petersen et al. 1995; Kloepper et al. 2004).

We isolated wild-type strains of two types from the soil and collected mutants of the typical *B. mycoides* colony morphotype, all characterized by looser cell connections. These mutants fall within a few classes that progressively modify the colony chiral shape. The most divergent is the round and compact colony of SINett, a SIN-derived phenotypic mutant whose mutation has not been determined. Light microscopy analysis of colony growth on agar showed that the cell chains of SINett are those most frequently interrupted during multiplication on the Petri dish surface (Di Franco et al. 2002). In the present study, in which we bound fluorescent vancomycin (Van-BDP) to live cells, we found that *B. mycoides* is characterized by the frequent polar localization of the antibiotic. The poles most frequently fluorescent were those identified in strains that typically formed non-hyphal compact colonies.

## Materials and methods

### Bacterial strains and growth conditions

*Bacillus mycoides* strains DX and SIN are environmental laboratory stocks, while SINett is a spontaneous phenotypic SIN mutant (Di Franco et al. 2002). *B. subtilis* subsp *subtilis* 168 (BGSC 1A1) was obtained from the Bacillus Genetic Stock Center, Columbus, Ohio. Bacilli were all cultured in TS broth (Trypton Soya, Oxoid) and the colonies formed on 15 g/l agar in TS. Growth temperature for *B. subtilis* was 37 °C. *B. mycoides* strains were grown at the optimal temperature of 30 °C, the duplication time being 25–30 min in these conditions. Overnight cultures were diluted about 100-fold in fresh medium and incubated 2–3 h so that cells could be collected in the exponential growth phase. Spores were prepared from old Petri dish cultures by vortexing strains in fresh TS broth and repeatedly discarding debris by centrifugation. The spore suspension was incubated in TS broth at 30 °C for 2–5 h until the majority of the spores germinated.

### Vancomycin staining

The MIC of the DX and SIN *B. mycoides* strains was detected at 2 μg/ml for BODIPY-FL conjugate vancomycin (Van-BDP, Molecular Probes) and at 0.2 μg/ml for unlabeled vancomycin (Sigma) in TS. The concentrations normally used in this study, blocking growth of all the before mentioned strains, were 2 μg/ml Van-BDP plus 2 μg/ml unlabeled vancomycin for 10 min at 30 °C (*B. mycoides*) and at 37 °C (*B. subtilis*) in the dark. Live cells grown to the mid-exponential phase or newly produced from germinated spores were treated and then washed with fresh TS broth, loaded on polylysine-treated microscopic slides and mounted in Vectashield (Vector laboratories), with the DNA-dye DAPI (4’, 6-diamidino-2-phenylindole) at a final concentration of 0.25 μg/ml.

### Live-cell fluorescence microscopy

Cells were examined with a Zeiss Axioplan microscope with an HBO 50 W mercury lamp for epifluorescence, equipped with a cooled charge-coupled device (Sensys). Greyscale digital images were collected separately with RS Image v1.9.2 software (Roper Scientific). CCD camera exposure times were set at Automatic Exposure: Intensity Target of 1,000 pxl and Upper Limit of 4 s. Images were converted to Photoshop vs 7.0 format (Adobe), pseudocolored and merged.

## Results

### Fluorescent vancomycin staining of *B. mycoides*

The distribution of PG precursors was analyzed in cells of the *B. mycoides* strains studied in our laboratory (Di Franco et al. 2002). We used the BODIPY derivative of the antibiotic vancomycin (Van-BDP), chosen on account of the small size of the fluorescent tag, which affords easier access to the cell wall D-Ala-D-Ala ligands and presents fewer aggregation problems than Van-FL (Tiyanont et al. 2006). The minimal inhibitory concentration of Van-BDP (MIC) on *B. mycoides* cells was found at 2 μg/ml, while the concentration of unlabeled vancomycin required to interrupt growth was lower, that is approx. 0.2 μg/ml. Staining of the cells was too faint when Van-BDP was used below the MIC. Increasing concentrations of Van-BDP were then tested, either alone or mixed with unlabeled vancomycin, which was reported to increase staining of the lateral cell wall in *B. subtilis* (Daniel and Errington 2003; Tiyanont et al. 2006). Addition of unlabeled vancomycin did not change the lateral wall fluorescence in *B. mycoides*, while it increased staining in *B. subtilis* as expected. The staining procedure thereafter routinely used a mixture 1:1 of Van-BDP and unlabeled vancomycin, both at 2 μg/ml, which worked well for both *B. mycoides* and *B. subtilis*. The antibiotics, when above the MIC, blocked cell growth and revealed the instant pattern of PG precursors binding the fluorescent probe. The exposition period to Van-BDP was usually about 10 min, but the cell decoration was not modified in experiments lasting from 1 min up to hours, the only consequence during long treatments being an increase in the number of cells that lost their cellular content and thus appeared as “ghosts”. The staining of the sidewall, septa and poles remained similar even in the ghosts.

**Fig. 2:**
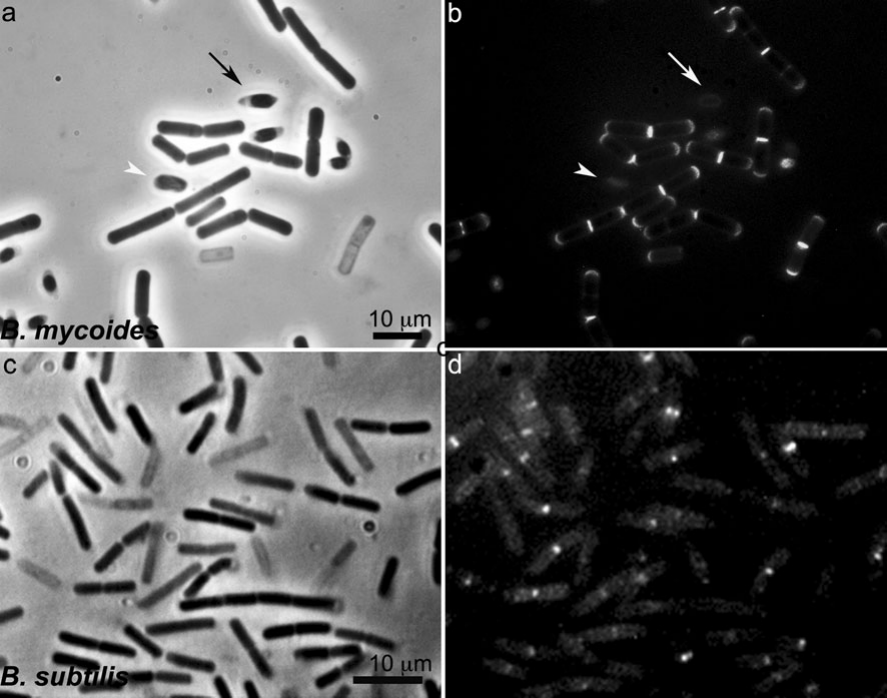
View of *B. mycoides* SIN and *B. subtilis* cells from germinated spores. The short filaments arose from spores seeded in TS broth. After 4 h at 30 °C (SIN) and at 37 °C (*B. subtilis*), Van-BDP/vancomycin at 2 μg/ml each was added for 10 min. **a** phase contrast and **b** Van-BDP fluorescence of *B. mycoides* SIN; **c** phase contrast and **d** Van-BDP fluorescence of *B.subtilis* 168. Note that the rod cells are mainly stained by Van-BDP at septa and poles in *B. mycoides*, and mainly at septa and along the lateral wall in large foci in *B. subtilis*. Some cells are “ghosts”, stained nonetheless by Van-BDP. The *arrow* points to a spore starting germination and the *arrowhead* to a spore in a more advanced germination stage

When we treated fresh unfixed cells of *B. subtilis* 168 and *B. mycoides* strains DX, SIN and SINett, the mutant with a non-hyphal colony (Fig. 1 top panel) with Van-BDP as described, fluorescence was very intense at midcell septa both in the *B. mycoides* strains and in *B. subtilis* 168 (Fig. 1b, arrows). Fluorescence was quite uniformly concentrated in small spots along the cylindrical cell body in *B. mycoides*, while *B. subtilis* 168 exhibited bright sidewall staining in large foci (Figs. 1, 2).

Unexpectedly, several *B. mycoides* cell poles displayed a consistent amount of fluorescence along the entire length of their curvature, while the poles in *B. subtilis* were generally unstained. Polar Van-BDP fluorescence was observed at the edges between separate cells that were connected and formed filaments (Fig. 1b, thick arrows) and at the free poles of filament edges (Fig. 1b, stars). Polar labeling was obtained by treating cells from exponential vegetative cultures (Fig. 1) and from germinated spores (Fig. 2). In cultures initiated from germinated spores (Fig. 2), the short filaments were all composed of newborn cells, and the poles could not be mistaken as septa of broken cells as might occur in cultures of vegetative cells. When cells grown on agar were also analyzed, they yielded the same results.

**Fig. 3:**
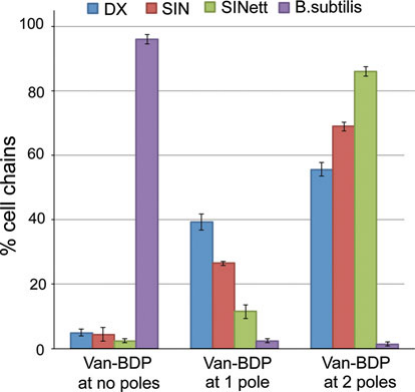
Van-BDP at the poles in *B. mycoides* strains and in *B. subtilis*. The histogram shows the percentage of the cell chains that show at the free external poles no Van-BDP binding, binding to only one pole or to both poles as the average of at least 3 independent experiments. Error bars show standard deviations. The poles of *B. subtilis* are very rarely bound by Van-BDP. Among the *B. mycoides* strains, Van-BDP is found more frequently at both poles in the mutant SINett

The transition from the midcell septum to the two new poles is a continuous process in dividing cells. The internal poles in a cell chain can then be easily confused with uncompleted septa. We thus stated the frequency of polar staining by Van-BDP considering in the statistics (Fig. 3) as bona fide poles only the free external poles of the cell chains.

Not all of the external free poles carried Van-BDP fluorescence. The frequencies were calculated as the average of several experiments, taking into account the values from not less than 90 filaments considered in each observation. As shown in the histogram of Fig. 3, in the DX strain, 39 % of the cell chains showed one of the two external poles stained by Van-BDP, 56 % had both the external poles stained and only 5 % of the chains did not show polar staining at all. In SIN, the values were: 27 % of the chains had one pole stained, 69 % had two poles stained and 4 % no poles stained. Both DX and SIN are characterized by very long cell chains that form the typical hairy colony (Fig. 1 top). In SINett, with a round compact colony made up by shorter filaments, both poles were stained more frequently. The values were: 12 % of the chains had one pole stained, 86 % had both poles stained and 2 % no poles stained. In *B. subtilis* 168, we found instead that 96 % of the chains had no poles stained. The latter finding is in agreement with classical experiments that indicate the polar regions of *B. subtilis* as relatively inert, with low wall turnover (Clarke-Sturman et al. 1989; Daniel and Errington 2003).

In the same experiments, DAPI staining evidenced that septa did not form at any potential division site, as demonstrated by the fact that two or more nucleoids without intervening septa in DX and SIN filaments were not uncommon (Fig. 1c, d). In SINett, by contrast, nucleoid separation and positioning of septa coexisted, as also occurred in the *B. subtilis* sample.

## Discussion

Vancomycin, the so-called last-resort drug for antibiotic-resistant infections, is a useful tool for detecting new PG location sites in the bacterial cell wall (Daniel and Errington 2003; Tiyanont et al. 2006). This antibiotic binds to the C-terminal D-Ala-D-Ala residues present in the pentapeptide of lipid II-linked disaccharides, the PG precursors, and in the un-crosslinked peptides of nascent peptidoglycan (Vollmer et al. 2008). When bound to a fluorescent tag, vancomycin decorates the cellular sites of massive PG synthesis, above all the division septa. It may also bind inside the old PG, where interpeptide bonds may not have been completed, leaving a free D-Ala-D-Ala group exposed, but the background in such cases is randomly distributed and not condensed into visible spots.

The localization of fluorescent vancomycin in fresh cells of *B. mycoides* strains revealed some characteristic features. The reactive PG along the cell sidewall, if compared with *B. subtilis*, was randomly distributed and formed smaller foci that do not appear to be arranged helically (Daniel and Errington 2003, Carballido-Lopez 2006). Recently, the helical arrangement of new PG synthesis sites was not confirmed in *B. subtilis* following the introduction of a more sensitive analysis method. Total internal reflection fluorescence microscopy (TIRFM) was used to follow the movement inside the live cell of GFP-linked actin-like proteins and GFP proteins of the multienzyme complexes for PG synthesis, showing GFP-proteins moving in a circular motion along radial bands perpendicular to the cell length (Garner et al. 2011, Dominguez-Escobar et al. 2011). These observations are consistent with the model for peptidoglycan architecture proposed for *B. subtilis* on the basis of atomic force microscope imaging (Hayhurst et al. 2008). In the cell body of *B. mycoides*, where the sites of PG neosynthesis are quite uniformly distributed, the enzyme complexes might be located along thinner radial bands.

Another characteristic of *B. mycoides* and of other bacilli of the same group (preliminary data not shown) is the persistent Van-BDP fluorescence at several poles after cell separation, which is in contrast to the rarely observed reaction to vancomycin of the free poles in *B subtilis*.

The possible reasons underlying polar labeling can as yet only be hypothesized. At the pole, after division and cell separation, new PG synthesis might continue, differently from *B. subtilis*, because it is required by a particular architecture of the pole.

Alternatively, no new PG synthesis is occurring, and polar labeling might be due exclusively to pools of free PG precursors, left over after the end of septation and slowly diluted away from the new pole. This explanation does not, however, hold for the polar staining observed in cells born from spores. Newly germinated 2-cell and 4-cell chains (see Fig. 2) have external poles that do not originate from a septation event but come directly from the spore and are, nonetheless, brightly labeled. Van-BDP fluorescence, which is low in non-germinated spores (see the spores indicated by the arrow and the arrowhead in Fig. 2), becomes intense at the poles, suggesting active recruitment of PG precursors at that location for new wall synthesis.

Active accumulation of PG precursors at a polar site may be required to prepare that precise cell edge for oriented cell multiplication (Dworkin 2009). When we observed growth on agar plates of the DX and SIN *B. mycoides* strains under the microscope, we noticed that the colonies extended in a centrifugal direction, mainly through multiplication of the cells at the filament edges. Polarized growth may thus be considered typical of these strains. Filament interruptions are likely to be downregulated in the hyphal strains like DX and SIN by growth inhibition at one cell pole, which would explain the higher number of cell chains with only one outer pole bound by Van-BDP compared to non-hyphal strains, that is the SINett mutant, which has both poles bound more frequently by Van-BDP (see histogram of Fig. 3). Non-polarized but simultaneous growth at adjacent poles of two consecutive cells may determine tensions within a filament causing the cell chain interruptions observed during colony construction in SINett (Di Franco et al. 2002).

Our data, which are based on cytological observations, highlight some interesting features of the cell wall in *B. mycoides*, shared with other members of the *B. cereus* group. We cannot yet use the various DNA transformation-based molecular biology tools that have shed light over the years on numerous processes in model bacteria, because the environmental *B. mycoides* DX and SIN strains carry multiple restriction activities against exogenous DNA. The homogeneous distribution of vancomycin binding in members of the *B. cereus* group will, however, make it easier to investigate polar PG synthesis in strains that lend themselves to DNA transformation.
